# Spontaneous Postpartum Rupture of an Intact Uterus: A Case Report

**DOI:** 10.14740/jocmr1922w

**Published:** 2014-10-16

**Authors:** George Mavromatidis, George Karavas, Chrysoula Margioula-Siarkou, Stamatios Petousis, Ioannis Kalogiannidis, Apostolos Mamopoulos, David Rousso

**Affiliations:** a The 3rd Department of Obstetrics and Gynecology, Aristotle University of Thessaloniki, Greece

**Keywords:** Uterine rupture, Unscarred uterus, Postpartum rupture, Vaginal delivery

## Abstract

Rupture of uterus is an obstetrical complication characterized by a breach in the uterine wall and the overlying serosa. We report an unusual case of spontaneous rupture of an unscarred uterus in a 33-year-old woman, a day after her third successful vaginal delivery. A 33-year-old pregnant woman, gravid 3, para 3, was referred to our department at 39 gestational week because of rupture of membranes. Despite tocolysis administration, her pregnancy was delivered vaginally after 2 days, giving birth to a male neonate of 3,020 g with normal Apgar scores at first and fifth minute. Her uterus was intact and gynecological examination after delivery was normal without any potential signs or symptoms of pathology. However, the day following her labor, patient complained of left iliac fossa pain. Her blood tests revealed a CRP value at 27.6 mg/L, whereas the X-rays revealed an extensive impacted fecal mass in the colon. MRI revealed that the left lower myometrial part of the uterus was depicted abrupt, with simultaneous presence of hemorrhagic stuff. The decision of laparotomy was therefore made in order to further evaluate rupture of uterus and properly treat patient. And subtotal hysterectomy was performed. Postoperative follow-up period was not characterized by any complications and patient was finally discharged 4 days after hysterectomy.

## Introduction

Rupture of uterus is an obstetrical complication characterized by a breach in the uterine wall and the overlying serosa. It occurs particularly during labor or third trimester of pregnancy and it is a hazardous condition to both maternal and fetal health. Postpartum hemorrhage, need for blood transfusion and hysterectomy, as well as high risk for neonatal peripartum death are of the most important consequences following uterine rupture [1, 2].

Uterine rupture is divided into two main categories: rupture in a scarred uterus and rupture in an intact uterus. The term “scarred uterus” is referred to the uterus of a woman that has previously undergone gynecological operations and predominantly cesarean section, which constitutes the principal cause of overall uterine ruptures [3-5]. Further factors contributing to this unfavorable situation are malpresentation, second stage dystocia, labor induction, preterm delivery, delivery after 42nd gestational week, multiparity, advanced maternal age, abnormal placentation, fetal macrosomia, multiple gestation, congenital uterine anomalies, instrumental delivery and external trauma [4-7].

Incidence of uterine rupture is affected by the level of medical care and the presence of scar in the uterus. Therefore, it is a very rare condition in the developed world and even more in women with unscarred uteri [1, 3]. According to literature, incidence of spontaneous rupture in a previously intact uterus is approximately 1 in 15,000 [8]. We report an unusual case of spontaneous rupture of an unscarred uterus in a 33-year-old woman, a day after her third successful vaginal delivery.

## Case Report

A 33-year-old pregnant woman was referred to our department at 39^th^ gestational week because of rupture of membranes. Concerning her past obstetrical history, she had two uncomplicated term vaginal deliveries, 10 and 8 years ago respectively. Regarding the obstetrical history of her present pregnancy, she referred first trimester bleeding, which was cured with progesterone per os. Moreover, vaginal culture of first trimester revealed Escherichia coli. After her admission in our department, 3 mg of alprostadil was administrated vaginally followed by 10 IU of oxytocin in 1 L Ringer’s lactate solution. Labor did not progress. After 2 days of simple hydration, we induced labor with the same therapeutic approach and labor was successfully and uneventfully completed vaginally after approximately 9 h. The neonate was a male of 3,020 g with normal Apgar scores at first and fifth minute.

**Figure 1:**
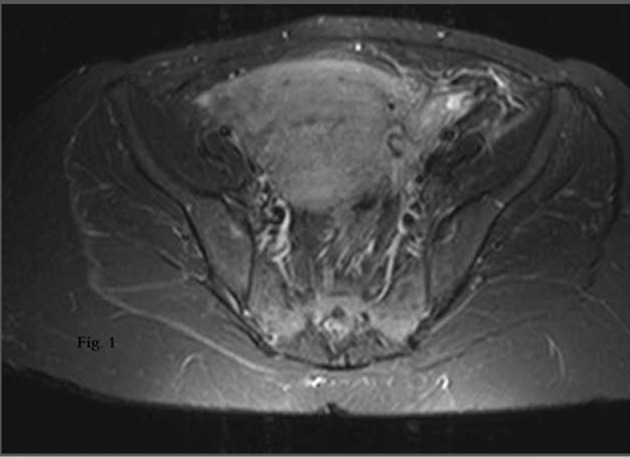
MRI of left lower myometrial part of the uterus: abruption with simultaneous presence of hemorrhagic stuff.

Gynecological examination after delivery was normal and did not indicate any signs or symptoms of pathology. However, the day following her labor, patient complained of left iliac fossa pain. Her blood tests revealed a CRP value at 27.6 mg/L, whereas the X-rays revealed an extensive impacted fecal mass in the colon. Colon evacuation did not lead to pain amelioration and the abdominal computed tomography (CT) that was performed 2 days after revealed filthiness of the left parametrium with pachynsis of peritoneum. CT was followed by magnetic resonance imaging (MRI), performed the next day. According to MRI, the left lower myometrial part of the uterus was depicted abrupt, with simultaneous presence of hemorrhagic stuff (Fig. 1). In parallel, an increase was observed in CRP values (CRP = 172 mg/L) and in her temperature (37.7 °C).

The decision of laparotomy was therefore made in order to further evaluate rupture of uterus and properly treat patient. The decision of subtotal hysterectomy was made as conservative surgical management was not possible due to the extension of rupture in left parametrium. Postoperative follow-up period was not characterized by any complications and patient was finally discharged 4 days after hysterectomy.

## Discussion

Spontaneous rupture of uterus is a rarely observed complication in modern obstetrical practice. Progress in prenatal care in combination with eligible management of women with previous cesarean deliveries has contributed to the decrease of uterine rupture. According to a systematic review conducted by World Health Organization (WHO), the median incidence of uterine rupture was estimated at 5.3 per 10,000 deliveries [9]. However, in developing countries, there is an eight-fold higher risk of uterine rupture due to the elevated rates of home deliveries and inadequate obstetrical care [1, 3].

Although antenatal identification of risk factors can reduce the possibility of uterine rupture by closer and careful management of the pregnancy, there have been reported cases of spontaneous rupture of the uterus without certain risk factors [10, 11]. However, there was no obvious risk factor for rupture concerning our patient’s case. A potential parameter that might partially offer a plausible explanation to the uterine rupture is the induction of labor. However, literature associates administration of oxytocin and prostaglandin agents particularly with intrapartum uterine rupture [1, 7, 8, 12]. Additionally, there is a discrepancy between authors regarding the role of labor induction in uterine rupture, as it is not recognized in unison as an independent factor for this obstetric complication [4, 13-15].

Advanced maternal age, multiparity and fetal macrosomia have been also mentioned to raise the possibility of uterine rupture [4, 6, 16]. Regarding the case which we presented, the patient was a relatively young woman and gave birth vaginally to her third child, a neonate with a normal weight of approximately 3,000 g. The majority of published articles outline the grand multiparity as a potential contributor to rupture of uterus [1, 4, 17]. Characteristically, Ripley reported a 20-fold higher risk of uterine rupture in women with at least seven previous deliveries [18]. Furthermore, in a recent national case-control study, Fitzpatrick et al showed that the subpopulation of women whose labor was complicated with uterine rupture had a parity of 3 or more [7]. However, there are authors claiming that multiparity is not positively correlated with spontaneous rupture of uterus [19, 20].

Uterine rupture in our patient could not be attributed to anyone of the certain risk factors. A parameter that might explain such an unusual postpartum rupture of the uterus is impaired collagen synthesis due to a potential underlying collagen deficiency or chronic steroid use [12]. Additionally, endometriosis, arteriovenus malformation and abnormal placentation have been accused to be associated with higher possibility of uterine rupture [5, 21]. None of the former risk factors, though, was confirmed in our case. The only reasonable explanation for the uterine rupture of our gravid might be an infection of the uterus, such as endometritis, which would offer in parallel a reason for the raised levels of CRP.

The most impressive aspect of our case is that the rupture appears to have occurred postpartum. To the best of our knowledge, this case of uterine rupture might be the only one in literature that occurred a day after completion of labor in a previously intact uterus. Another case of postpartum uterine rupture has also been reported. However, it is referred to a postpartum rupture of a uterus after cesarean delivery [21]. Although it could be assumed that the rupture in our patient might have occurred intrapartum or shortly after delivery, her clinical symptoms do not stand for this possibility. Clinical presentation of peripartum uterine rupture includes impaired fetal activity, vaginal bleeding, abdominal pain, maternal tachycardia and other symptoms of hypovolemia [7, 21]. On the contrary, the woman appeared to have a normal postpartum period devoid of any problems. Characteristically, there was no complaint on anything, her vital signs were normal and stable, and she walked uneventfully and even proceeded to breastfeeding.

In conclusion, uterine rupture is a complication in obstetrics that is difficult to be predicted due to its unclear and plural etiopathogenesis. Despite its rare occurrence, an obstetrician should have this complication in mind especially in cases with certain risk factors and be aware of the difficulty in its diagnosis due to potential nonspecific signs and symptoms.
